# The emerging role of autophagy in Parkinson's disease

**DOI:** 10.1186/1756-6606-2-29

**Published:** 2009-09-16

**Authors:** Zelda H Cheung, Nancy Y Ip

**Affiliations:** 1 Department of Biochemistry, Molecular Neuroscience Center and Biotechnology Research Institute, Hong Kong University of Science and Technology, Clear Water Bay, Kowloon, Hong Kong, PR China

## Abstract

Parkinson's disease (PD) is the most common neurodegenerative movement disorder that affects about 1% of the population worldwide. Despite significant advances in the identification of genetic mutations and signaling pathways that are associated with the disease, the precise mechanisms implicated in the pathophysiology of the disease are not well understood. More importantly, treatments that are effective in reversing the progression of the disease is essentially lacking. Further investigation into the pathogenic mechanisms of PD thus presents a pressing concern for neuroscientists. Recently, deregulation of the autophagic pathway is observed in the brains of PD patients and in models of PD. In this review we summarize current literature on the emerging involvement of autophagy in PD, and the implication for future development of treatment against the disorder.

## Introduction

Parkinson's disease is a neurodegenerative disorder characterized by the degeneration of neurons in the substantia nigra pars compacta of the brain. On a cellular level, neuronal loss is accompanied by neurite degeneration and the presence of cytoplasmic inclusions known as Lewy bodies. PD is the most common neurodegenerative movement disorder and the second most common neurodegenerative disease, with the prevalence rising from 1% at age 65 to about 4-5% at age 85 [1]. Most of the PD cases are sporadic, although familial PD with autosomal dominant or autosomal recessive mutations also account for about 5% of all PD cases [2]. PD patients suffer from resting tremor, bradykinesia, muscle rigidity and postural instability. While the deterioration of motor functions observed in PD patients is predominantly attributable to the degeneration of dopaminergic neurons in the substantia nigra, neuron loss in other brain regions, including locus ceruleus and raphe nucleus, are also observed in post-mortem brains of PD patients [3].

Despite the prevalence of the disease, current treatment only ameliorates symptoms by attempting to reverse dopaminergic deficits. Extensive effort has therefore been directed at explicating the pathogenic mechanisms of PD, in hope of identifying targets that would enable reduction of neuronal loss and halting of disease progression. Various pathogenic mechanisms have been associated with neuronal loss in PD, including oxidative stress, mitochondrial dysfunction, protein aggregation and abnormality with the ubiquitin-proteasome pathway [3]. In particular, identification of autosomal dominant and autosomal recessive mutations in cases of familial PD has significantly contributed to our current understanding of the pathogenesis of PD. For example, autosomal dominant A53T and A30P mutation of alpha-synuclein, the main constituent of Lewy bodies, is associated with familial PD [4,5]. Interestingly, mutant alpha-synucleins are more prone to oligomerization [6]. Furthermore, increased gene dosage of alpha-synuclein is associated with certain cases of familial PD [7,8]. These observations point to a pivotal involvement of alpha-synuclein and protein aggregation in the pathogenesis of PD. Presence of protein aggregates in PD brains could also be attributed to deregulation of the ubiquitin-proteasome degradation system. Mutations in Parkin, an ubiquitin E3-ligase, and ubiquitin-C-terminal hydrolase-L1 (UCH-L1), a protein that is involved in the degradation of poly-ubiquitin chains, are both associated with familial PD cases [9,10]. On the other hand, evidence in support of a role of mitochondrial dysfunction in PD came from the identification of mutations in PINK1 and Parkin, both of which have been demonstrated to regulate mitochondrial morphology and function [11,12].

Interestingly, recent studies reveal that deregulation of autophagy is evident in the brains of PD patients [3]. Indeed, the autophagic pathway is increasingly implicated in a number of neurodegenerative diseases [13]. In the following sections, we summarize existing knowledge on the role of autophagy in PD.

## The autophagic pathway

**Figure 1:**
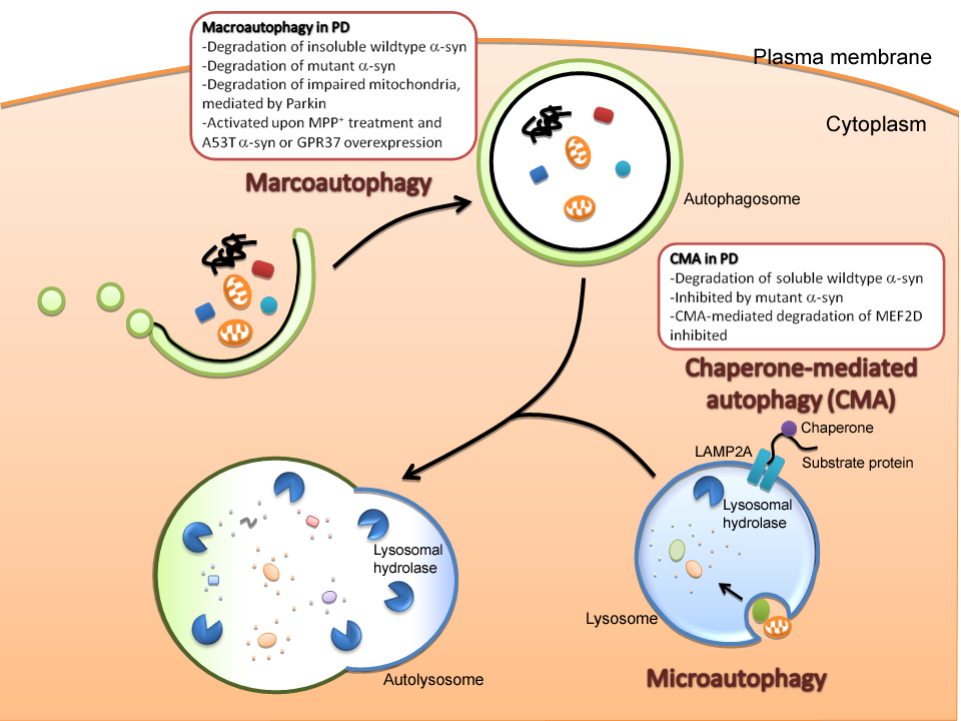
**Implication of autophagy deregulation in Parkinson's disease (PD)**. There are 3 types of autophagy: macroautophagy, microautophagy and chaperone-mediated autophagy (CMA). See text for detailed description of the different types of autophagy. Deregulation of both macroautophagy and CMA are implicated in the pathogenesis of PD. CMA is involved in the degradation of soluble wildtype alpha-synuclein, the major constituent of Lewy bodies. Nonetheless, once oligomerized or aggregated, alpha-synuclein is likely degraded by macroautophagy instead of CMA. Interestingly, A53T and A30P mutants of alpha-synuclein are poorly degraded by CMA, but are instead degraded by macroautophagy. Furthermore, the alpha-synuclein mutants inhibit CMA, reducing CMA-mediated degradation of alpha-synuclein and survival factor MEF2D. Concomitant with the inhibition of CMA, overexpression of alpha-synuclein mutants results in a compensatory activation of macroautophagy. Activation of macroautophagy is also evident in cells treated with neurotoxin MPP^+^, a well-established model for Parkinsonism, or following overexpression of GPR37, another protein that is present in Lewy bodies. Finally, recent studies reveal that macroautophagy also plays a role in the turnover of fragmented mitochondria. These observations highlight the potential involvement of the autophagic pathways in the pathogenesis of PD.

Autophagy is traditionally considered as a cellular homeostatic process pivotal for bulk degradation of cytoplasmic contents and organelles. Aside from the ubiquitin-proteasome degradation pathway, which is involved mainly in the breakdown of short-lived proteins, the autophagic pathway represents the other major mechanism by which intracellular proteins are degraded. The autophagic pathway is responsible for the degradation of long-lived proteins, protein aggregates and cytoplasmic organelles [14]. Degradation of proteins and organelles begin with the sequestration of cargo into various vesicles of the autophagic pathway. Based on the mechanisms by which cargos enter the autophagic pathway, 3 different forms of autophagy have been identified: macroautophagy, microautophagy and chaperone-mediated autophagy (CMA). Macroautophagy is probably the best characterized form of autophagy among the three. Sequestration of cargo begins with the formation of a double-membraned structure known as the autophagosome. Cargos are either sequestered through bulk engulfment of the cytoplasmic content, or through selective targeting. Content of the autophagosomes are then degraded through fusion with lysosomes, leading to the formation of autolysosomes [13,15]. Microautophagy is similar to macroautophagy in that cargos enter the autophagic pathway through direct sequestration of cellular contents, but cargos are sequestered directly into the lysosomes instead of the autophagosomes [13]. On the other hand, CMA is distinct from macroautophagy and microautophagy in several aspects. First of all, instead of sequestration of cargo through bulk engulfment of cytoplasmic contents, proteins destined for CMA usually contain a targeting motif KFERQ and are selectively translocated into the autophagic pathway. Secondly, CMA is predominantly involved in the degradation of soluble proteins. Proteins targeted for CMA are recognized by cytosolic chaperone heat-shock cognate 70 (Hsc70), followed by transportation directly into the lysosomes through association with lysosomes-associated membrane protein 2A (LAMP2A) [13,14]. Figure 1 summarizes the various types of autophagy.

## Deregulation of the autophagic pathway in PD

Given the role of the autophagic pathway as a degradation system for clearance of aggregated proteins, it is not surprising that autophagic deregulation observed in various neurodegenerative disorders has attracted mounting interests. For example, accumulation of autophagosomes are evident in the brains of Alzheimer's disease patients [13]. Indeed, protein aggregates presents one of the common features in a myriad of neurodegenerative diseases, including the β-amyloid plagues in Alzheimer's disease, mutant huntingtin cytoplasmic inclusions in Huntington's disease, and the alpha-synuclein containing Lewy bodies in PD. While the role of these protein aggregates in the pathogenesis of these disorders remain controversial, it is plausible that removal of protein aggregates through activation of the autophagic pathway would interfere with the potential toxicity of these aggregates, thereby alleviating disease progression. Indeed, accumulating evidence supports a protective role of autophagy through clearance of protein aggregates. For example, reduction of huntingtin aggregates in a model of Huntington's disease through activation of macroautophagy limits neuronal loss and behavioral deficits [16]. Furthermore, knock down of autophagy-related (ATG) genes, molecular players that are pivotal for the induction and execution of macroautophagy, results in neurodegeneration and the presence of cytoplasmic inclusions filled with ubiquitinated proteins [17,18]. These observations suggest that basal level of autophagy is important for the clearance of protein aggregates, and the reduction of cytoplasmic inclusions may be protective against neurodegenerative diseases. Nonetheless, excessive activation of the autophagic pathway has also been associated with cell death [15]. It is therefore pivotal to delineate the precise involvement of autophagy in the pathogenesis of neurodegenerative diseases.

### Autophagy as a protective mechanism in PD

One of the first evidence in support of an important role of autophagy in PD came from the demonstration that alpha-synuclein is degraded by macroautophagy and CMA [19-21]. Since alpha-synuclein is the major constituent of Lewy bodies, which is a pathological hallmark in both sporadic and familial cases of PD, it is important to delineate the mechanisms by which alpha-synuclein is degraded. Interestingly, while the ubiquitin-proteasome pathway and macroautophagy are both implicated in the clearance of alpha-synuclein, CMA was found to be essential for the degradation of wildtype alpha-synuclein [19-21]. Furthermore, inhibition of CMA results in the formation of high molecular weight and detergent-insoluble species of alpha-synuclein [21], revealing that in healthy neurons, clearance of alpha-synuclein by CMA is crucial for limiting the oligomerization of alpha-synuclein. Importantly, A53T and A30P mutants of alpha-synuclein, which cause familial PD, were found to inhibit CMA through exhibiting a higher affinity for LAMP2A [20]. Nonetheless, alpha-synuclein mutants are poorly internalized into lysosomes, and are degraded by macroautophagy instead of CMA. Accompanying the inhibition of CMA by alpha-synuclein mutants is a compensatory activation of macroautophagy, although the physiological significance of this event is incompletely understood. Nonetheless, these observations suggest that A53T and A30P alpha-synuclein mutants may induce alpha-synuclein aggregation through inhibition of CMA, thereby leading to impaired clearance of cellular alpha-synuclein. On the other hand, 193M missense mutation in UCH-L1, which was previously identified in familial cases of PD, is also implicated in the regulation of CMA [22]. They found that mutant UCH-L1 exhibits an abnormally high affinity towards key players in the CMA pathway, such as LAMP2A and Hsc70. More importantly, overexpression of UCH-L1 mutant increases alpha-synuclein level [22], suggesting that UCH-L1 mutation may also contribute to PD pathology through regulating alpha-synuclein levels. These findings reveal that inhibition of CMA-mediated degradation of alpha-synuclein may constitute an important pathogenic mechanism for PD.

Interestingly, it was recently demonstrated that alpha-synuclein may also contribute to neuronal death in PD through inhibiting CMA-mediated degradation of survival factor MEF2D [23]. Both wildtype and A53T mutant of alpha-synuclein interfere with the binding of MEF2D to CMA machinery Hsc70. Consistent with this observation, MEF2D level is elevated in A53T alpha-synuclein transgenic mice, and also in PD patients. Furthermore, overexpression of both wildtype and A53T mutant of alpha-synuclein inhibit MEF2 activity, and result in neuronal death [23]. These observations reveal that in addition to alpha-synuclein, impaired CMA may also trigger neuronal death through inefficient degradation of other proteins. These findings collectively raise the interesting possibility that preservation of CMA functions may serve as an effective treatment against PD.

Aside from the degradation of alpha-synuclein, the autophagic pathway is also involved in the turnover of mitochondria. Mitochondrial dysfunction presents one of the pathogenic mechanisms in PD. In particular, deficits in the mitochondrial complex I is observed in PD patients. Furthermore, MPTP, the neurotoxin that generates PD symptoms in human through selective degeneration of dopaminergic neurons in the substantia nigra, functions as a complex I inhibitor after it is converted into MPP^+ ^[3]. More importantly, mutations identified from cases of familial PD have been mapped to Parkin and PINK1, two proteins that are implicated in the control of mitochondrial morphology and function [11,12]. Mutation in these two proteins presumably leads to loss of function, thus resulting in mitochondrial abnormality. Recently, Parkin was found to facilitate macroautophagy of impaired mitochondria, a process that is also known as mitophagy [24]. Parkin is selectively targeted to mitochondria that are impaired, characterized by a low mitochondrial transmembrane potential, which are then eliminated by macroautophagy [24]. In support of a role of autophagy in the clearance of defective mitochondria in PD, knock down of PINK1 expression induces mitochondrial fragmentation, followed by activation of autophagy/mitophagy [25]. These observations suggest that the autophagic pathway is essential for the turnover of dysfunctional mitochondria in PD. Failure to activate efficient mitophagy may serve as a pathogenic mechanism of PD.

### Could excessive activation of the autophagic pathway lead to cell loss?

Despite mounting observations that are in support of a protective role of autophagy in various models of PD, one essential question remains unanswered. While it is perceivable that insufficient autophagy activation would impair clearance of protein aggregates and dysfunctional mitochondria, whether excessive activation of autophagy happens in PD, and whether it plays a role in PD pathology remains unknown. This question is particularly important since excessive activation of autophagy is associated with neuronal loss [15]. Indeed, abnormal presence of autophagic vacuoles is evident in the brains of PD patients, in contrast to the rare detection of autophagosomes in normal brain, due to the rapid clearance of these vesicles in the central nervous system [26,27]. The presence of autophagosomes in PD brains could represent aberrant activation of macroautophagy, although defective clearance of the autophagic vacuoles could also account for this observation. While there is little evidence to delineate the two possibilities, *in vitro *findings reveal that macroautophagy can be induced by players associated with PD pathology. For example, as mentioned previously, overexpression of alpha-synuclein mutants results in activation of macroautophagy [20,21]. Aside from alpha-synuclein, GPR37, another protein that is present in Lewy bodies, is also recently demonstrated to induce macroautophagy [28]. Furthermore, treatment with MPP^+ ^has also been observed to increase autophagic vacuole contents [29]. Clearly, a question that urgently needs to be addressed is whether the induction of macroautophagy results in cell death. Several studies provided evidence that overexpression of A53T mutant of alpha-synuclein in PC12 cells, cultured neurons and the nigrostriatal systems results in autophagic cell death [23,30-32]. These studies indicate that while activation of macroautophagy may serve as a protective machinery, excessive activation could result in neuronal death and hence also contribute to cell loss in PD.

## Conclusion

Given the lack of effective treatment targeted at halting disease progression and neuronal loss in PD, advances in the development of novel therapeutic strategy would rely on a better understanding of the pathogenic mechanisms of PD. Mounting evidence suggests that the autophagic pathway is emerging as a key pathway for regulating the pathogenesis of PD. Nonetheless, as mentioned above (Figure 1), whether activation of autophagy serves a beneficial or detrimental role in PD remains controversial. Since autophagy is part of the cell's homeostatic machinery, basal autophagic activity would undoubtedly be important for minimizing abnormal protein aggregation and for facilitation of organelle turnover. Maintaining autophagic activity at a certain level is particularly important since the level of macroautophagy and CMA are both observed to decrease with age [33]. Development of therapeutic agents that boost autophagic response could thus serve as a strategy to combat protein aggregation in PD, and also in other neurodegenerative diseases with cytoplasmic inclusions. Nonetheless, since excessive activation of the autophagic pathway leads to cell death, designing a drug that would only activate autophagy to a certain optimal extent would be essential, albeit extremely challenging. Ultimately, more physiologically relevant evidence on the activation status of the autophagic pathway in PD patients would be required to determine if activation or suppression of autophagy would be most effective in ameliorating PD symptoms and delaying disease progression. Future studies aiming at resolving the existing controversy would be pivotal in turning these findings into effective treatment against PD.

## Abbreviations

ATG: autophagy-related; CMA: chaperone-mediated autophagy; Hsc70: heat-shock cognate 70; LAMP2A: lysosomes-associated membrane protein 2A; PD: Parkinson's disease; UCH-L1: ubiquitin-C-terminal hydrolase-L1.

## Competing interests

The authors declare that they have no competing interests.

## Authors' contributions

ZC and NI wrote, read and approved the final manuscript.
